# Evaluation of Hormonal Factors in Acne Vulgaris and the Course of Acne Vulgaris Treatment with Contraceptive-Based Therapies in Young Adult Women

**DOI:** 10.3390/cells11244078

**Published:** 2022-12-16

**Authors:** Dominika Borzyszkowska, Mirela Niedzielska, Mateusz Kozłowski, Agnieszka Brodowska, Adam Przepiera, Kinga Malczyk-Matysiak, Aneta Cymbaluk-Płoska, Elżbieta Sowińska-Przepiera

**Affiliations:** 1 Department of Reconstructive Surgery and Gynecological Oncology, Pomeranian Medical University in Szczecin, Al. Powstańców Wielkopolskich 72, 70-111 Szczecin, Poland; 2 Department of Endocrinology, Metabolic and Internal Diseases, Pomeranian Medical University in Szczecin, Unii Lubelskiej 1, 71-252 Szczecin, Poland; 3 Department of Gynecology, Endocrinology and Gynecological Oncology, Pomeranian Medical University in Szczecin, Unii Lubelskiej 1, 71-252 Szczecin, Poland; 4 Department of Urology and Urologic Oncology, Pomeranian Medical University in Szczecin, Al. Powstańców Wielkopolskich 72, 70-111 Szczecin, Poland; 5 Pediatric, Adolescent Gynecology Clinic, Department of Gynecology, Endocrinology and Gynecological Oncology, Pomeranian Medical University in Szczecin, Unii Lubelskiej 1, 71-252 Szczecin, Poland

**Keywords:** acne, acne vulgaris, hormonal factors, contraception, treatment, testosterone, androstendione, SHBG, FAI

## Abstract

Acne vulgaris is a common chronic inflammatory skin disease, which is considered one of the diseases of civilization due to the significant influence of environmental factors on the severity and frequency of these lesions. The aim of this study was to evaluate the hormonal profile of patients before treatment and to assess selected hormonal parameters after treatment. Our first objective was to examine the correlation between the selected hormonal parameters and the severity of acne before treatment. Our second objective was to evaluate the impact of treatment with three therapies, as measured by the selected hormonal parameters and acne severity. Statistical calculations were performed using the R v.4.1.1 statistical calculation environment (IDE RStudio v. 1.4.1717) with a significance level for the statistical tests set at α = 0.05. The results showed that the women in the pre-treatment (T1) and control (C) groups had significant differences in testosterone, androstendione, FAI, SHBG, prolactin, ACTH, and cortisol concentrations. After treatment, there were still significant differences in testosterone, androstendione, FAI, and SHBG concentrations between the post-treatment (T2) and control groups. We concluded that testosterone, androstendione, and cortisol concentrations correlate with acne severity. Acne in adult women may be an important clinical marker of androgen excess syndrome and cannot be considered a transient symptom of puberty. The mainstay of acne treatment is contraceptive therapy (ethonylestradiol and drospirenone). In this study, we confirmed the effectiveness of three contraceptive-based treatments using hormonal parameters and acne severity.

## 1. Introduction

Acne vulgaris (AV) is a common chronic inflammatory skin disease that is considered one of the diseases of civilization due to the significant influence of environmental factors on the severity and frequency of these lesions [1]. The condition affects about 9.4% of the world’s population. It is usually associated with adolescence but can occur between the ages of 11 and 30. AV is particularly prevalent in late adolescence, between the ages of 15 and 18, when it is most likely initiated by the onset of puberty [2]. It is believed to affect 80% of people in this age group and up to 100% of young people [3,4,5]. The dermatosis usually appears in the second decade of life, then becomes less severe with age, and finally resolves at the end of the second or the beginning of the third decade of life. However, there are cases where the disease process has persisted until the third, and even beyond the fourth, decade of life [6]. Acne that occurs in women aged >25 is defined as adult female acne, which may differ from the juvenile type in its specific clinical aspects and evolution and in its propensity for chronicity [7]. Various studies have found that the prevalence of acne in adult women ranges from 12 to 54% and that it is more common in women compared to men [8]. Acne lesions are 95% localized on the face and upper torso, but they can also occasionally occur on other parts of the body. Due to the localization of the lesions and the chronic nature of the condition, AV is often a serious psychological problem for teenage girls and young women [9,10,11].

The skin in the pathogenetic understanding of acne consists of three layers: epidermis, dermis, and subcutaneous tissue [12]. The epidermis is mainly made up of maturing epithelial cells called keratinocytes. An important role of keratinocytes is their ability to produce hormones and respond via receptors to peripherally circulating hormones. The dermis consists of skin appendages including the sweat glands, sebaceous glands, nails, and hair. Acne lesions form on the sebaceous gland. Sebaceous glands consist of a single or a group of lobules [13]. Hormones synthesized by endocrine glands exert well-characterized pleiotropic regulatory effects on various skin cell types due to the expression of cytoplasmic membrane-bound or nuclear receptors with a high affinity for the hormones involved [14,15]. Androgen receptors (AR) are expressed in the cytoplasm of various skin cell types, including keratinocytes, sebocytes, fibroblasts, endothelial cells, sweat gland cells, and dermal papilla cells. The binding of androgens to AR induces several biological effects in various skin cell populations, such as the regulation of sebaceous gland function by controlling sebaceous cell proliferation and sebum secretion [16]. Studies have shown that androgens in the skin cause an attenuation of the inflammatory process in cells that have high levels of AR, such as keratinocytes, macrophages, neutrophils, monocytes, and T cells. The direct inhibitory effect of androgens on inflammation arises from the activation of overlapping signalling pathways, mainly the ERK-1 or ERK-2 pathway, which ultimately modulate the expression and activity of inflammatory mediators, including the cytokines TNF-α and IL-1β as well as the C-reactive protein [17,18]. It has also been shown that increasing the androgen/estrogen ratio can exacerbate the severity of acne in patients with acne vulgaris [19]. In addition, sebaceous gland activity is influenced by the growth hormone, insulin, cortisol, prolactin, thyroid hormones, and adrenaline. Excess amounts of the growth hormone cause abnormal lipogenesis and excessive sebum secretion. Elevated insulin levels stimulate sebocyte proliferation. Cortisol causes an increase in sebaceous gland activity, and elevated levels of cortisol in stressful situations are manifested by increased serum (sebum) secretion and the exacerbation of acne lesions. Prolactin causes an increase in androgen levels and, through this mechanism, an increase in sebum secretion. Thyroid hormones and adrenaline increase lipogenesis in sebocytes, resulting in an increased amount of sebum being produced, which is why the skin condition worsens with chronic stress [20]. In cases affected by AV, high levels of circulating androgens excessively stimulate the proliferation of sebaceous cells and keratinocytes, interfere with the differentiation and exfoliation of keratinocytes, and promote lipid synthesis by sebaceous cells, leading to excessive seborrhea. Thus, the group of hormones most involved in the development of acne lesions are considered androgens. Testosterone is metabolized to dihydrotestosterone (DHT) by 5-alpha reductase and to 17-beta estradiol by aromatase. Thus, the skin can be considered a peripheral endocrine gland [21,22].

It is believed that the basic principle of acne treatment is to act on pathogenic factors. Hormonal treatments for acne include (1) anti-androgens (e.g., spironolactone, flutamide, and anti-androgenic progestogens including desogestrel, drospirenone, and cyproterone acetate) and (2) blocking the ovarian and/or adrenal androgen production (e.g., estrogens, cyproterone acetate, gonadotropin-releasing hormone (GnRH) agonists, low doses of glucocorticosteroids, and oral contraceptives) [23]. However, it has been found that hirsutism and menstrual irregularity can occur during treatment with oral isotretinoin [24]. Oxybrasion [25] and hydrogen purification [26] have also been used in the treatment of acne vulgaris.

The aim of this study was to evaluate the hormonal profile of patients before treatment and to assess the selected hormonal parameters after treatment. The first objective was to examine the correlation between selected hormonal parameters and acne severity before treatment. The second objective was to evaluate the impact of treatment with three therapies, as measured by the selected hormonal parameters and acne severity.

## 2. Materials and Methods

### 2.1. Participants and Selection Criteria

#### 2.1.1. Study Groups and Treatment Regimens

The study included 168 women aged 18–31 years who were patients of the Gynecologic Endocrinology Outpatient Clinic at the Department of Endocrinology, Metabolic and Internal Diseases at the Pomeranian Medical University in Szczecin and other dermatology outpatient clinics. The subjects were divided into two groups: the study group, including 99 patients with acne vulgaris, and the control group, including 69 patients without skin lesions. The study group was divided into the following subgroups according to the treatment used:

- Subgroup OC was treated with an oral contraceptive agent,
- Subgroup OC + A was treated with an oral contraceptive agent + cyproterone acetate,
- Subgroup OC + R was treated with an oral contraceptive agent + isotretinoin.

All patients in the study group received a contraceptive formulation with the following composition: 0.03 µg ethonylestradiol + 3 mg drospirenone. The patients were then classified into one of the three subgroups described above: OC, OC + A, or OC + R.

The OC + A group received an additional 50 mg of cyproterone acetate 1 × 1 for 10 days per treatment cycle for 3 months. When a reduction in acne severity was achieved, the cyproterone acetate dose was halved and continued for another 3 months.

The OC + R group was treated with isotretinoin at an initial dose of 10 mg for 3 months. This treatment only achieved a partial reduction in the severity of acne. Therefore, the isotretinoin treatment was continued with a dose of 20 mg for another 3 months.

The pre-treatment (T1) patients were defined as the patients before the administration of acne therapy. The post-treatment (T2) patients were defined as the patients after 6 months of follow-up.

#### 2.1.2. Inclusion Criteria for the Study

The general inclusion criteria for the study included:

- Age 18–31 years;
- Caucasian women;
- A history of normal sexual maturation;
- No history of permanent medication use;
- No significant abnormalities on physical examination;
- The patient consents to participate in the study.

The eligibility criteria for the study group included the presence of acne in young women who received no previous treatment. The control group included healthy young women without acne who attended the outpatient clinic for a preventive examination and consented to biochemical tests.

The criteria for exclusion from the study group included:

- Disorders of growth and weight gain;
- Endocrine diseases (e.g., thyroid disease, diabetes mellitus, polycystic ovary syndrome (PCOS), congenital adrenal hyperplasia (CAH), premature expiration of ovarian function (POF)), which were diagnosed based on history, gynecological examination, and laboratory tests;
- Performance of competitive sports;
- Long-term use of stimulants;
- Incomplete follow-up period;
- Application of a different acne treatment regimen.

### 2.2. Instruments

All patients underwent a subjective and physical examination. The following anthropometric measurements were obtained: height (cm) and weight (kg). The body mass index (BMI [kg/m^2^]) of each patient was calculated using these measurements.

The hormonal parameters were determined from fasting venous blood collected in the morning. The following hormone concentrations were obtained: androstendione, dihydroepiandrosterone sulfate (DHEA-S), testosterone (T), sex hormone binding protein (SHBG), luteinizing hormone (LH), folliculotropic hormone (FSH), prolactin (PRL), ACTH, and cortisol. Based on the results, the free androgen index (FAI) was calculated. Hormonal tests were performed between days 3 and 5 of the menstrual cycle. The tests were performed in the laboratory using the following standard methods: electrochemiluminescence-luminescence assay (ECLIA), immunoenzymatic assay (ELISA), chemiluminescence-immunoassay (CLIA), and calorimetric assay.

Visual assessment based on the LEEDS scale was used to evaluate acne [27].

### 2.3. Statistical Methods

Statistical calculations were carried out using the R v.4.1.1 statistical computing environment (IDE RStudio v. 1.4.1717). The significance level of the statistical tests was α = 0.05. For variables on an interval scale, grouped descriptive statistics were used to obtain a description of the study set and to draw some basic conclusions and generalizations about the samples. In addition, a normality test was conducted based on the Shapiro–Wilk test including the W test statistic and an indication of *p* significance.

The hypotheses of the Shapiro–Wilk test include:

**H0:**  *The sample does not come from a population with a normal distribution.*

**H1:**  *The sample comes from a population with a normal distribution.*

The variables on a nominal, ordinal scale were analyzed in pairs using contingency tables with an indication of frequency. The relationship of the variables was examined using Fisher’s exact test, and Cramer’s V measures of relationship strength were calculated.

The hypotheses of Pearson’s chi-square test and Fisher’s exact test include:

**H0:**  *the variables are independent, and there was no relationship between the two nominal variables.*

**H1:**  *the variables are dependent, and there was a relationship between the two nominal variables.*

For independent variables with a normal distribution and more than two independent groups, Welch’s one-way analysis of variance (ANOVA) was used to test the significance of differences. For two groups of independent samples with a normal distribution, the Welch’s *t*-test with Hedges’ g effect size calculation was used. For two groups of independent samples with a non-normal distribution, the Mann–Whitney U test was used with the calculation of two-point correlation based on ranks (${\hat{r}}_{biserial}^{rank}$). For two dependent variables with a normal distribution, the Student’s *t*-test with Hedges’ g effect size calculation was used to test the significance of differences. For two dependent variables with a normal distribution, the non-parametric Wilcoxon paired rank-sum test was used. The value of the association between the variables was calculated using the rank-based two-point correlation (${\hat{r}}_{biserial}^{rank}$). When calculating the correlation between variables on the ordinal scale and the quotient scale, the Kendall rank correlation coefficient τb was calculated in the form of an appropriate measure of the relationship. When calculating the correlation between a variable on an ordinal scale and a variable on a nominal (dichotomous) scale in the form of a correlation coefficient, a two-point correlation based on ranks was estimated (${\hat{r}}_{biserial}^{rank}$). When the ordinal variable had only two levels, Yule’s phi linkage measure (φc) was used.

### 2.4. Ethics

This study was conducted in accordance with the Declaration of Helsinki and approved by the Ethics Committee of the Pomeranian Medical University in Szczecin (protocol code KB-012/78/18 on 18 June 2018).

## 3. Results

### 3.1. Descriptive Statistics of Variables

The study group and the control group were not statistically significantly different in terms of the study variables, as shown in Table 1.

The women in the pre-treatment (T1) and control (C) groups showed significant differences in testosterone, androstendione, FAI, SHBG, prolactin, ACTH, and cortisol concentrations. After treatment (T2), there were still significant differences in the values of testosterone, androstendione, FAI, and SHBG concentrations, as shown in Table 2.

### 3.2. Relationships between Hormonal Factors and Acne Severity before Treatment

Significant correlations were found between testosterone levels and the LEEDS scale (*p* = 0.013), between androstendione levels and the LEEDS scale (*p* = 0.014), and between cortisol levels and the LEEDS scale (*p* = 0.017). There was borderline significance between ACTH and the LEEDS scale (*p* = 0.051). The remaining correlations were not significant. The detailed correlations between the studied hormones and the LEEDS scale are shown in Table 3.

### 3.3. The Significance of Differences between Selected Parameters in Subjects from the Pre- and Post-Treatment Group by the Type of Agents Used

#### 3.3.1. Assessment of Testosterone Levels before and after Treatment

##### Treatment Type: Oral Contraceptive (OC)

For the treatment with an oral contraceptive, the Student’s *t*-test for the dependent variables showed a significant reduction in testosterone levels from M = 0.48 ng/mL, SD = 0.15 ng/mL before treatment to M = 0.37 ng/mL, SD = 0.12 ng/mL after treatment (*t_Student_*(33) = 5.38, *p* < 0.001). The resulting effect was estimated as “large”, ${\hat{g}}_{Hedges}$ = 0.9.

##### Treatment Type: Oral Contraceptive (OC) + Cyproterone Acetate (A)

For the treatment with an oral contraceptive and cyproterone acetate formulation, the Student’s *t*-test for the dependent variables showed a significant reduction in testosterone levels from M = 0.66 ng/mL, SD = 0.14 ng/mL before treatment to M = 0.42 ng/mL, SD = 0.13 ng/mL after treatment (*t_Student_*(30) = 8.98, *p* < 0.001). The resulting effect was estimated as “large”, ${\hat{g}}_{Hedges}$ = 1.57.

##### Treatment Type: Oral Contraceptive (OC) + Isotretinoin (R)

For the treatment with an oral contraceptive formulation and an isotretinoin formulation, the Student’s *t*-test for the dependent variables showed a non-significant reduction in testosterone levels from M = 0.48 ng/mL, SD = 0.21 ng/mL before treatment to M = 0.44 ng/mL, SD = 0.16 ng/mL after treatment (*t_Student_*(33) = 1.49, *p* < 0.145). The resulting effect was estimated as “small”, ${\hat{g}}_{Hedges}$ = 0.25.

**Figure 1:**
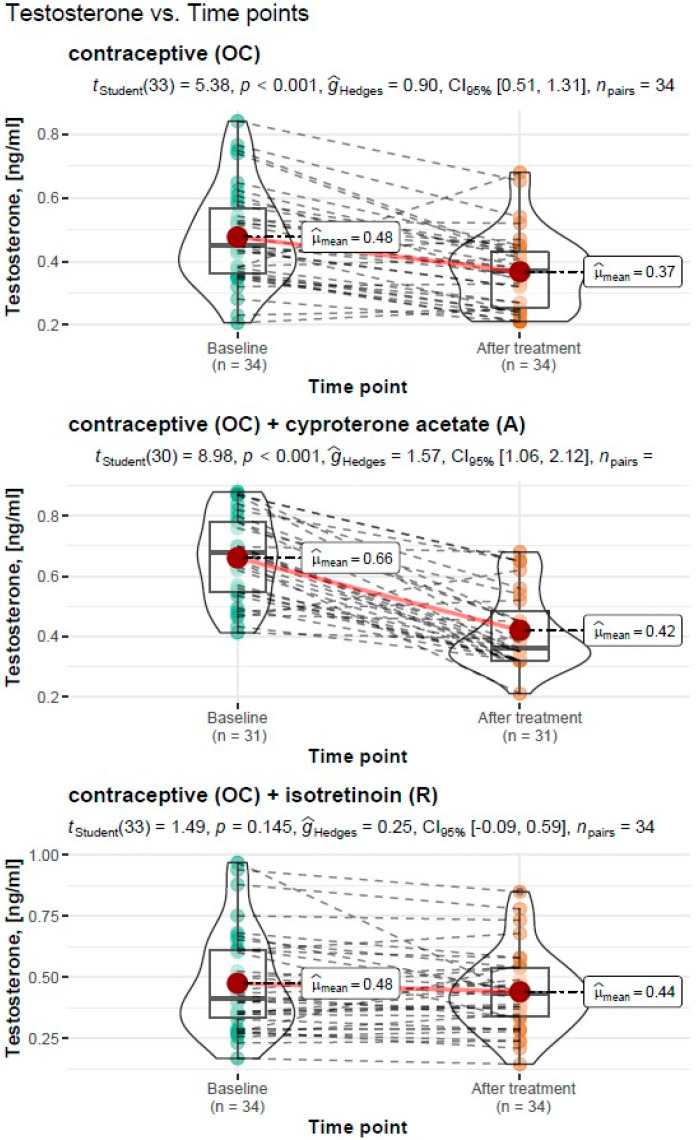
Comparison of mean testosterone levels before and after treatment by the type of treatment carried out, with reporting of the results of the statistical test.

A graphical representation of the comparison of the mean testosterone levels and the expanded statistical test reporting by treatment type is shown in Figure 1.

#### 3.3.2. Assessment of Androstendione Levels before and after Treatment

##### Treatment Type: Oral Contraceptive (OC)

For the treatment with an oral contraceptive, the Student’s *t*-test for the dependent variables showed a significant reduction in androstendione levels from M = 3.48 ng/mL, SD = 0.98 ng/mL before treatment to M = 2.81 ng/mL, SD = 0.70 ng/mL after treatment (*t_Student_*(33) = 6.98, *p* < 0.001). The resulting effect was estimated as “large”, ${\hat{g}}_{Hedges}$ = 1.17.

##### Treatment Type: Oral Contraceptive (OC) + Cyproterone Acetate (A)

For the treatment with an oral contraceptive formulation and cyproterone acetate formulation, the Student’s *t*-test for the dependent variables showed a significant reduction in androstendione levels from M = 4.67 ng/mL, SD = 0.78 ng/mL before treatment to M = 3.29 ng/mL, SD = 0.64 ng/mL after treatment (*t_Student_*(30) = 14.37, *p* < 0.001). The resulting effect was estimated as “large”, ${\hat{g}}_{Hedges}$ = 2.52.

##### Treatment Type: Oral Contraceptive (OC) + Isotretinoin (R)

For the treatment with an oral contraceptive formulation and an isotretinoin formulation, the Student’s *t*-test for the dependent variables showed a significant reduction in androstendione levels from M = 3.53 ng/mL, SD = 0.78 ng/mL before treatment to M = 3.00 ng/mL, SD = 0.64 ng/mL after treatment (*t_Student_*(33) = 6.98, *p* < 0.001). The resulting effect was estimated as “large”, ${\hat{g}}_{Hedges}$ = 1.17.

**Figure 2:**
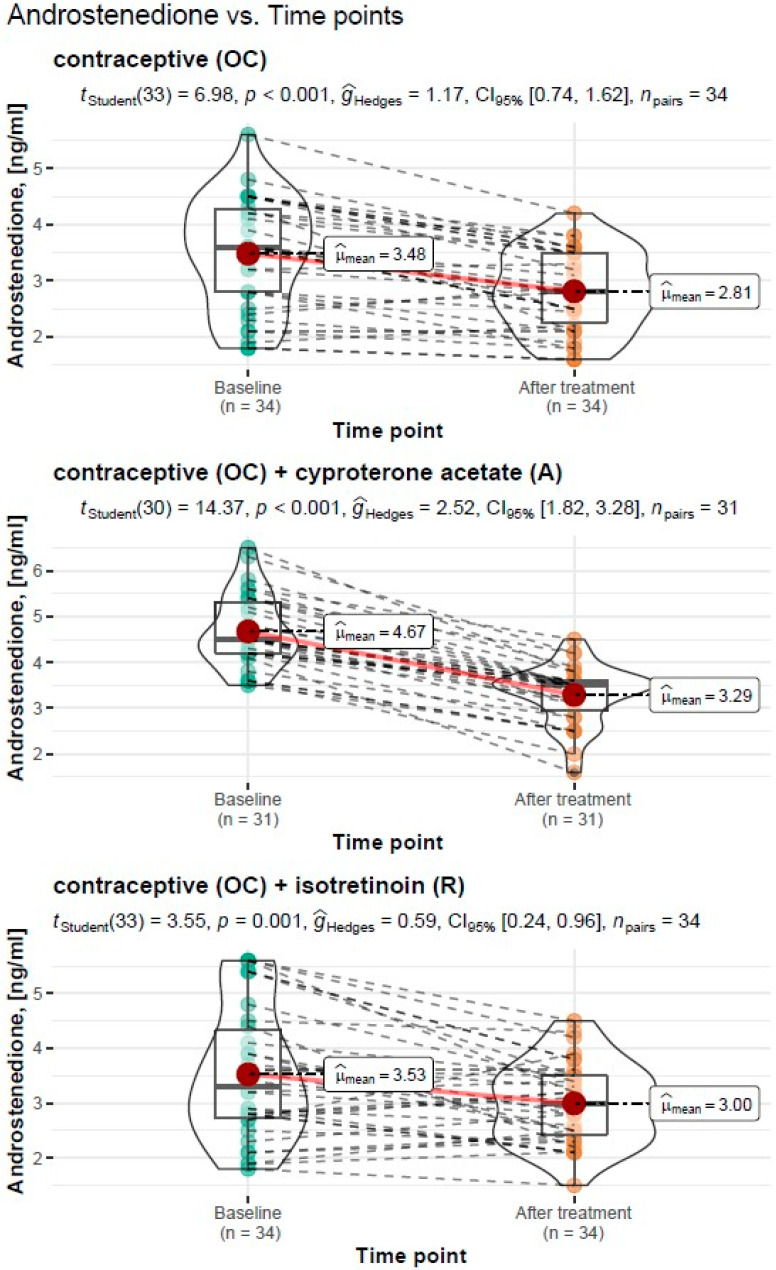
Comparison of mean androstendione concentrations before and after treatment by type of treatment carried out with reporting of statistical test results.

A graphical representation of the comparison of mean androstendione levels and the expanded statistical test reporting by treatment type is shown in Figure 2.

#### 3.3.3. Assessment of SHBG Levels before and after Treatment

##### Treatment Type: Oral Contraceptive (OC)

For the treatment with an oral contraceptive, the Student’s *t*-test for the dependent variables showed a significant increase in the sex hormone binding globulin (SHBG) from M = 51.04 nmol/l, SD = 24.60 nmol/l before treatment to M = 138.12 nmol/l, SD = 35.20 nmol/l after treatment (*t_Student_*(33) = −11.65, *p* < 0.001). The resulting effect was estimated as “large”, ${\hat{g}}_{Hedges}$ = −1.95.

##### Treatment Type: Oral Contraceptive (OC) + Cyproterone Acetate (A)

For the treatment with an oral contraceptive formulation and cyproterone acetate formulation, the Student’s *t*-test for the dependent variables showed a significant increase in the sex hormone binding globulin (SHBG) from M = 47.68 nmol/l, SD = 20.0 before treatment to M = 173.47 nmol/l, SD = 40.2 nmol/l after treatment (*t_Student_*(30) = −18.50, *p* < 0.001). The resulting effect was estimated as “large”, ${\hat{g}}_{Hedges}$ = −3.24.

##### Treatment Type: Oral Contraceptive (OC) + Isotretinoin (R)

For the treatment with an oral contraceptive formulation and an isotretinoin formulation, the Student’s *t*-test for the dependent variables showed a significant increase in the sex hormone binding globulin (SHBG) from M = 39.39 nmol/l, SD = 16.10 nmol/l before treatment to M = 134.90 nmol/l, SD = 30.20 nmol/l after treatment (*t_Student_*(33) = −19.21, *p* < 0.001).The resulting effect was estimated as “large”, ${\hat{g}}_{Hedges}$ = −3.22.

**Figure 3:**
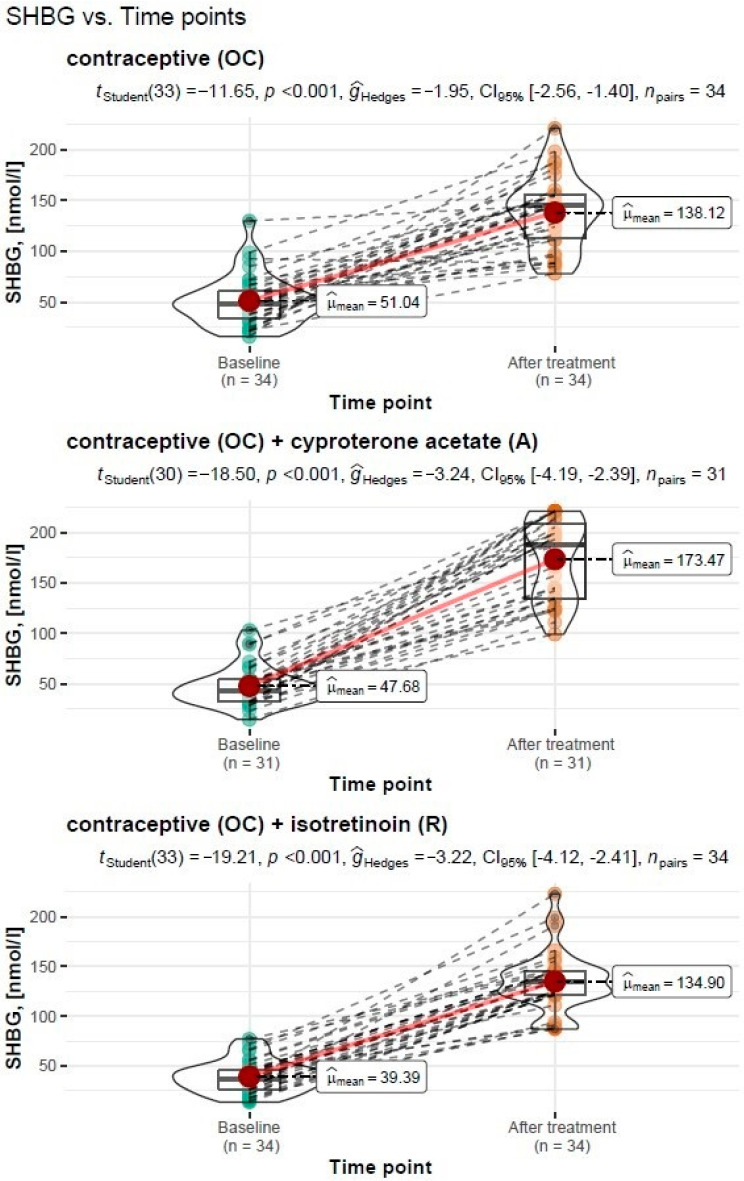
Comparison of mean SHBG levels before and after treatment by type of treatment carried out, with reporting of statistical test results.

A graphical representation of the comparison of mean the sex hormone binding globulin levels and the expanded statistical test reporting by treatment type is shown in Figure 3.

#### 3.3.4. Assessment of FAI before and after Treatment

##### Treatment Type: Oral Contraceptive (OC)

For the treatment with an oral contraceptive, the Wilcoxon paired rank-sum test for the dependent variables showed a significant reduction in the free androgen index (FAI) from Mdn = 1.03, IQR = 0.57 before treatment to Mdn = 0.14, IQR = 0.11 after treatment (*V_Wilcoxon_* = 595.00, *p* <0.001). The measure of association was estimated as “large”, ${\hat{r}}_{biserial}^{rank}$ = 1.0.

##### Treatment Type: Oral Contraceptive (OC) + Cyproterone Acetate (A)

For the treatment with an oral contraceptive formulation and cyproterone acetate formulation, the Wilcoxon paired rank-sum test for the dependent variables showed a significant reduction in the free androgen index (FAI) from Mdn = 1.45, IQR = 1.01 before treatment to Mdn = 0.23, IQR = 0.11 after treatment (*V_Wilcoxon_* = 496.00, *p* < 0.001). The measure of association was estimated as “large”, ${\hat{r}}_{biserial}^{rank}$ = 1.0.

##### Treatment Type: Oral Contraceptive (OC) + Isotretinoin (R)

For the treatment with an oral contraceptive formulation and the isotretinoin formulation, the Wilcoxon paired rank-sum test for the dependent variables showed a significant reduction in the free androgen index (FAI) from Mdn = 1.12, IQR = 1.01 before treatment to Mdn = 0.31, IQR = 0.13 after treatment (*V_Wilcoxon_* = 595.00, *p* < 0.001). The measure of association was estimated as “large”, ${\hat{r}}_{biserial}^{rank}$ = 1.0.

**Figure 4:**
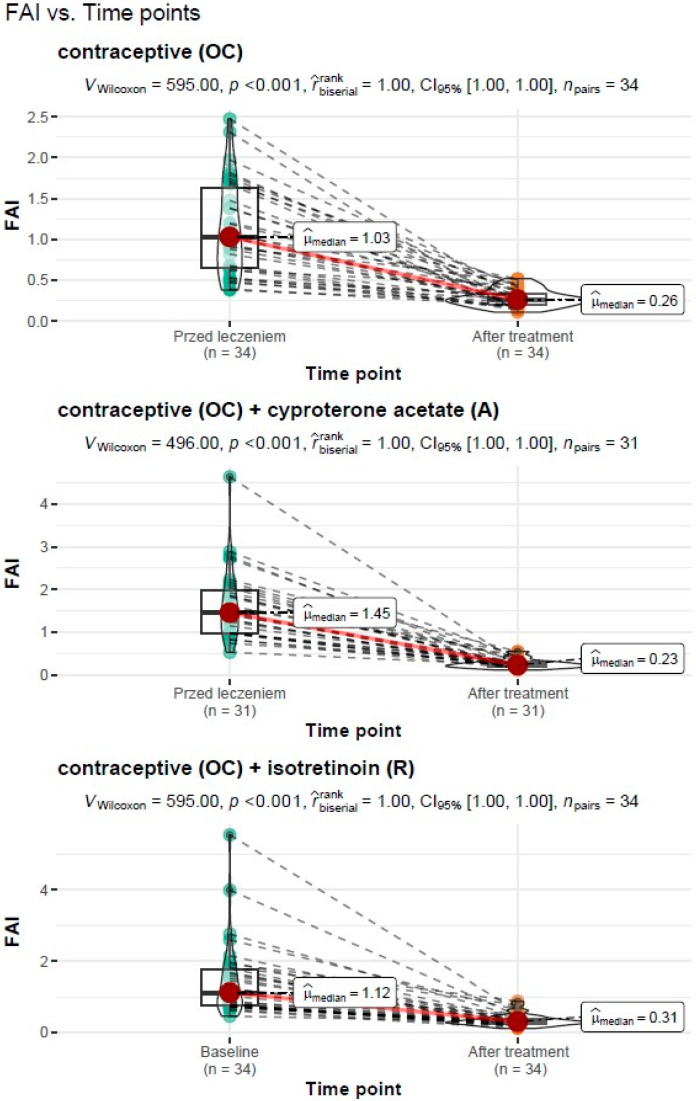
Comparison of the median free androgen index (FAI) before and after treatment by type of treatment carried out with reporting of statistical test results.

A graphical representation of the comparison of the median free androgen index and the expanded statistical test reporting by treatment type is shown in Figure 4.

#### 3.3.5. LEEDS Acne Severity Scores before and after Treatment

##### Treatment Type: Oral Contraceptive (OC)

For the treatment with an oral contraceptive, the Wilcoxon paired rank-sum test of the dependent variables showed a significant reduction in the severity of acne on the LEEDS scale from Mdn = 3.00, IQR = 0.00 before treatment to Mdn = 1.00, IQR = 0.00 after treatment (*V_Wilcoxon_* = 561.00, *p* < 0.001). The measure of association was estimated as “large”, ${\hat{r}}_{biserial}^{rank}$ = 1.0.

##### Treatment Type: Oral Contraceptive (OC) + Cyproterone Acetate (A)

For the treatment with an oral contraceptive and cyproterone acetate, the Wilcoxon paired rank-sum test for the dependent variables showed a significant reduction in the free androgen index (FAI) from Mdn = 1.45, IQR = 1.01 before treatment to Mdn = 0.23, IQR = 0.11 after treatment (*V_Wilcoxon_* = 496.00, *p* < 0.001). The measure of association was estimated as “large”, ${\hat{r}}_{biserial}^{rank}$ = 1.0.

##### Treatment Type: Oral Contraceptive (OC) + Isotretinoin (R)

For the treatment with an oral contraceptive formulation and the isotretinoin formulation, the Wilcoxon paired rank-sum test for the dependent variables showed a significant reduction in the free androgen index (FAI) from Mdn = 3.00, IQR = 1.00 before treatment to Mdn = 1.00, IQR = 2.00 after treatment (*V_Wilcoxon_* = 528.00, *p* < 0.001). The measure of association was estimated as “large”, ${\hat{r}}_{biserial}^{rank}$ = 1.0.

**Figure 5:**
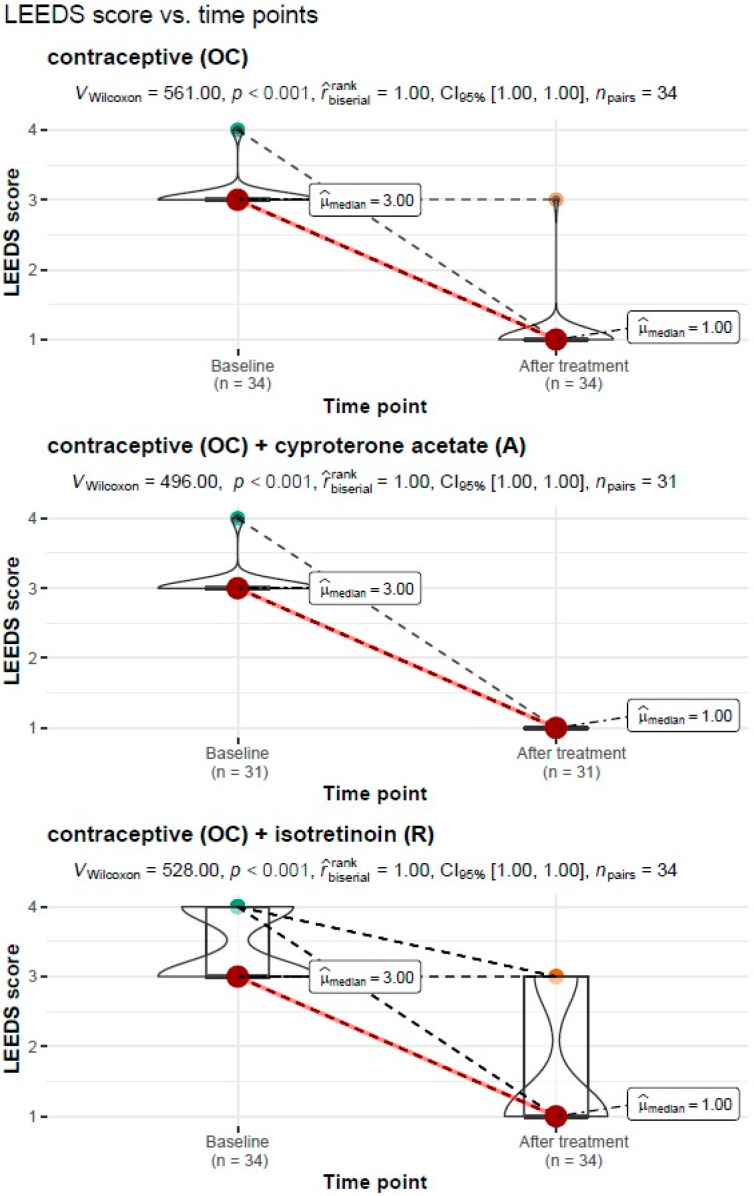
Comparison of the median acne severity on the LEEDS scale before and after treatment by the type of treatment carried out, with reporting of statistical test results.

A graphical representation of the comparison of the median acne severity on the LEEDS scale and the expanded statistical test reporting by treatment type is shown in Figure 5.

## 4. Discussion

Many groups have conducted research on the association of acne incidence with risk factors and lesion severity. Most previous studies have evaluated the relationship of AV incidence with a single clinical, metabolic, or hormonal parameter [28,29,30,31]. The pathogenesis of acne in adult women is particularly complex, so correlating several parameters simultaneously may prove more useful in clinical practice [32,33,34]. In this study, women in the pre-treatment group showed significantly higher values for androgen concentrations (testosterone, androstendione), FAI, prolactin, ACTH, and cortisol compared to the control group. After treatment, women in the post-treatment group still had higher values for testosterone concentrations, free androgen index FAI, and the sex hormone binding protein SHBG. Thus, androgenic stimulation of the sebaceous glands is an important factor in the development of acne. Similarly, Walton et al. conducted a study of 36 women (aged 14–34) and found a significant correlation between the number of acne lesions and some of the five androgenic hormone determinants such as free testosterone, androstendione, the sex hormone binding globulin (SHBG), dehydroepiandrostenedione sulfate (DHEAS), and dihydrotestosterone (DHT). This study also analyzed the prevalence of PCOS in women presenting to a dermatologist and found that the syndrome was diagnosed in only 19% of women with acne-like skin problems [35]. However, in a contradictory study, the authors found no positive correlation between acne severity and any of the clinical or laboratory markers of androgenization that were assessed. On the contrary, these authors found lower free testosterone index values and higher SHBG levels in women with higher acne severity. These results suggested that the severity of acne symptoms in adult women is not determined by androgen production alone. Moreover, only 19 women in the study group were diagnosed with PCOS [36]. Other authors evaluated the relationship between clinical symptoms and hormonal parameters in 129 women over 17 years of age with acne. They measured serum levels of ovarian- and adrenal-derived androgens and found elevated levels of at least one of the androgens tested in most patients, whereas only 19% had polycystic ovary syndrome. They also found that hirsutism and acne severity correlated negatively with the serum sex hormone binding globulin (SHBG) levels. In conclusion, the authors suggested that in post-adolescent women, acne severity appears to depend on peripheral hyperandrogenism, with a negative correlation between acne severity and serum SHBG levels [37]. In our study, we also found that the SHBG values were significantly lower in acne patients compared to the control group. The explanation for these results is that low serum SHBG values do not bind to produced testosterone in sufficient concentrations. This ultimately results in a higher concentration of the free form of the hormone, which affects the strength of its binding to androgen receptors and promotes the development of acne lesions [38]. Genetic factors may play a role in the synthesis and/or metabolism of the SHBG, conditioning relatively insufficient synthesis, excessive catabolism, or synthesis of forms with lower affinity for hormone receptors [39,40]. The results of our study also provide an explanation that the therapeutic effect of oral contraceptives on acne is realized, among other things, by increasing the SHBG production in the liver, thereby reducing the bioavailability of active testosterone.

The fact that acne depends on the action of androgens on the skin is confirmed by the clinical response in adult women to hormone therapy, especially in the context of disorders associated with hyperandrogenization such as PCOS syndrome. The use of hormonal therapies in the form of oral contraceptives and anti-androgen drugs in these women with androgen concentrations in the reference range results in a significant reduction in the severity of skin lesions (acne and seborrhea) [41]. This relationship is confirmed by the absence of acne in women with complete androgen insensitivity and with hereditary 5-alpha-reductase deficiency or with reduced dihydrotestosterone (DHT) production [42]. Other reports have evaluated the association of hyperandrogenemia with acne severity and hirsutism in women with PCOS syndrome and found that acne severity is not linearly related to androgen concentrations. The authors suggest that androgen concentrations should not be used to determine the dose of anti-androgen therapy, whereas the observed negative correlation between the SHBG concentrations and acne severity suggests that hormonal contraception, which increases the SHBG concentrations, should be used as primary therapy in these patients [34]. These studies completely agree with our results showing significant improvements in acne severity in all study models where OC was used. Thus, the relationship between androgen excess and the development of acne in adult women is well documented in studies by many authors [28,43,44,45,46]. In most cases, this excess androgen begins in adolescence, whereas persistent adult female acne (beginning in adolescence and persisting into adulthood) is more associated with hyperandrogenism than adult female acne beginning later in life [47]. An attempt to systematize research in this area was made by an international group of researchers who produced a report by the Multidisciplinary Committee on Hyperandrogenism and PCOS (AE-PCOS Society). Their thesis suggested that increased androgen production plays a major role in causing sebum changes that are involved in the pathogenesis of adult acne [48].

In our study, the pre-treatment group showed significant differences in the concentrations of androgens, prolactin, ACTH, and cortisol compared to the control group. Thus, the question arises whether prolactin could be another sebotropic hormone and whether it influences sebum secretion. Prolactin is a hormone whose excess can lead to the development of androgenization because in high concentrations it stimulates the production of androgenic hormones. Studies have shown that human skin within the hair follicle is also an extrapituitary source of prolactin and thyrotropin-releasing hormone (TRH) [49,50]. Given that stress is a source of excess prolactin and thyroid hormones, the presence of receptors for these hormones in the skin may be a direct and indirect cause of excess sebum and sweat gland secretion, which would result in increased acne. The nervous system communicates with peripheral tissues through nerve fibers and the systemic release of hypothalamic and pituitary neurohormones. Neuroregulation of the sebaceous glands (SG), the main appendage of the skin, is under close control of the pituitary gland and represents an interesting, clinically relevant, and peripheral target organ. Moreover, excess circulating growth hormone, thyroxine, or prolactin has been shown to cause increased sebum production (seborrhea). Conversely, growth hormone deficiency, hypothyroidism, and adrenal insufficiency cause decreased sebum production and dry skin [51,52]. In the human sebocyte (SG), the presence of specific receptors, e.g., for the adrenocorticotropic hormone, can stimulate sebum production independently of the gonads or adrenal glands, further emphasizing the importance of neuroendocrine control in SG biology [52]. These results are corroborated by the clinically known facts of more severe symptoms of sebum secretion by CRH and ACTH receptor stimulation of sebocytes. In addition, there is increased androgen secretion during ACTH-dependent Cushing’s syndrome compared to the case of adrenal adenoma, which is probably related to the simultaneous stimulation by ACTH of the adrenal cortex cells responsible for adrenal steroidogenesis and excessive secretion of DHEA and testosterone [53,54]. The observed correlations should signal the need for more in-depth knowledge on this topic in young adult women with acne.

Our study also had limitations. The main limitation was the six-month observation period which, from the perspective of the preparations used, did not allow us to assess the long-term effect of acne treatment in the subgroups. However, we are continuing the study to identify these long-term effects. Another limitation may be related to deficiencies in the obtained results, which is the reason for the different number of variables in some correlations. Some of the patients did not complete all the recommended test results. However, the missing values were for side parameters that did not form the core of this work, so despite this, we decided to exclude patients from the analysis. The patients’ use of several local potential determinants affecting acne severity and the LEEDS scale scores cannot be excluded. Nevertheless, due to the appropriate choice of statistical methodology and the large sample size, our findings are reliable and the assumptions are supported by their substantial consistency with published evidence. There were also concerns about the existing measures of acne severity and the inadequacy of existing acne severity scales. To address this concern, we conducted a critical review of the originally published acne scales to formally assess their quality based on a set of predetermined criteria. The lack of an internationally accepted measure of acne severity hinders high-quality clinical trials and the adoption of best practices with potential consequences for the acne sufferer. We conclude as others have, that a robust scoring system for assessing acne severity is required. Future development of acne scales or further study of currently available scales is needed to correct these problems and move closer to developing a valid and reliable “gold standard” tool.

## 5. Conclusions

Testosterone, androstendione, and cortisol levels correlate with the severity of acne. Acne in young adult women can be an important clinical marker of androgen excess syndrome but cannot be considered a transient symptom of puberty. The mainstay of acne treatment is contraceptive therapy (ethonylestradiol and drospirenone). The effectiveness of three contraceptive-based treatments was confirmed by hormonal parameters and acne severity.

## Figures and Tables

**Table 1:** Characteristics of study participants including age, weight, height, and BMI.

Variable	Group	*n*	M	SD	Mdn	IQR	Min	Max	*p*
Age	Study	99	25.93	5.95	25.00	9.00	18.00	31.00	0.6122
	Control	69	23.84	1.15	24.00	1.00	21.00	28.00	
Weight	Study	99	67.29	14.02	63.00	12.50	54.00	89.00	0.6688
	Control	69	60.46	7.06	60.00	9.00	58.00	85.00	
Height	Study	99	163.07	13.13	164.00	8.50	158.00	173.00	0.1949
	Control	69	164.58	5.45	164.00	8.00	155.00	176.00	
BMI	Study	99	25.00	5.00	24.00	5.05	18.69	30.72	0.5312
	Control	69	22.28	1.94	22.31	2.34	18.42	28.40	

N—sample size; M—mean; SD—standard deviation; Mdn—median; IQR—interquartile range; Min—minimum; Max—maximum; *p*—*p*-value.

**Table 2:** Detailed statistical characteristics of hormonal parameters in women in the study group (pre-treatment (T1) and post-treatment (T2)) compared to the control group (C).

Variable	Group	*n*	M	SD	Mdn	IQR	Min	Max	*p*
testosterone (T1)	Study	99	0.53	0.19	0.52	0.30	0.17	0.97	0.001
	Control	69	0.31	0.10	0.32	0.13	0.13	0.54	
testosterone (T2)	Study	99	0.41	0.14	0.38	0.14	0.14	0.85	0.001
androstendione (T1)	Study	99	3.87	1.14	3.90	1.60	1.80	6.50	0.001
	Control	69	2.36	0.58	2.47	0.73	1.19	3.83	
androstendione (T2)	Study	99	3.03	0.71	3.10	1.00	1.50	4.50	0.026
SHBG (T1)	Study	99	45.99	20.97	44.80	21.92	13.55	129.90	0.001
	Control	69	104.79	94.83	66.90	48.70	5.00	414.00	
SHBG (T2)	Study	99	148.08	38.94	145.10	55.70	78.10	223.10	0.001
FAI (T1)	Study	99	1.40	0.86	1.20	0.99	0.38	5.55	0.001
	Control	69	0.60	1.22	0.40	0.36	0.05	10.22	
FAI (T2)	Study	99	0.30	0.14	0.27	0.17	0.11	0.88	0.001
LH (T1)	Study Control	99 69	8.26 9.31	3.88 5.22	8.50 10.2	4.26 5.11	1.07 1.02	15.10 14.3	0.621
FSH (T1)	Study	99	5.63	1.81	5.47	2.51	1.64	9.95	0.820
	Control	69	5.61	1.84	5.82	2.53	0.30	9.69	
PRL (T1)	Study	99	20.66	7.99	19.60	13.03	6.75	37.91	0.001
	Control	69	17.91	8.33	16.30	8.60	6.16	44.00	
DHEA-S (T1)	Study	99	258.51	81.79	254.10	119.15	45.60	413.00	0.348
	Control	69	239.70	98.19	211.00	151.00	58.40	487.00	
ACTH (T1)	Study	99	16.57	11.95	15.20	13.35	4.27	89.20	0.001
	Control	69	21.21	11.52	18.70	12.90	1.66	67.90	
cortisol (T1)	Study	99	22.25	9.61	21.00	11.90	8.02	49.10	0.001
	Control	69	15.79	7.51	14.30	9.30	5.63	39.50	

T1—pre-treatment; T2—post-treatment; N—sample size; M—mean; SD—standard deviation; Mdn—median; IQR—interquartile range; Min—minimum; Max—maximum; *p*—*p*-value.

**Table 3:** Correlation coefficients between hormonal factors and acne severity on the LEEDS scale determined before treatment.

Variables	LEEDS Scale	
	Correlations (τ_b,_ φ_c_)	*p*
testosterone	0.14	0.013
androstendione	0.15	0.014
SHBG	0.07	0.369
FAI	0.05	0.531
LH	0.10	0.243
FSH	0.01	0.996
PRL	0.01	0.874
DHEA-S	0.01	0.870
ACTH	0.18	0.051
cortisol	0.20	0.017

## Data Availability

The data presented in this study are available on request from the author E.S.-P. The data are not publicly available due to ethical restrictions.

## References

[1] Chen H., Zhang T., Yin X., Man J., Yang X., Lu M. (2022). Magnitude and temporal trend of acne vulgaris burden in 204 countries and territories from 1990 to 2019: An analysis from the Global Burden of Disease Study 2019. Br. J. Dermatol..

[2] Lynn D., Umari T., Dellavalle R., Dunnick C. (2016). The epidemiology of acne vulgaris in late adolescence. Adolesc. Health Med. Ther..

[3] Tan J., Zhang X., Jones E., Bulger L. (2013). Correlation of photographic images from the Leeds revised acne grading system with a six-category global acne severity scale. J. Eur. Acad. Dermatol. Venereol..

[4] Tuchayi S.M., Makrantonaki E., Ganceviciene R., Dessinioti C., Feldman S.R., Zouboulis C. (2015). Acne vulgaris. Nat. Rev. Dis. Prim..

[5] Rao A., Douglas S., Hall J. (2021). Endocrine Disrupting Chemicals, Hormone Receptors, and Acne Vulgaris: A Connecting Hypothesis. Cells.

[6] Dréno B. (2017). What is new in the pathophysiology of acne, an overview. J. Eur. Acad. Dermatol. Venereol..

[7] Preneau S., Dreno B. (2012). Female acne—A different subtype of teenager acne?. J. Eur. Acad. Dermatol. Venereol..

[8] Dréno B. (2015). Treatment of adult female acne: A new challenge. J. Eur. Acad. Dermatol. Venereol..

[9] Koo J.Y.M., Smith L.L. (1991). Psychologic Aspects of Acne. Pediatr. Dermatol..

[10] Kantor J. (2022). This month in JAAD International: March 2022: The psychological impact of acne scarring. J. Am. Acad. Dermatol..

[11] Heng A.H.S., Chew F.T., Heng A.H.S., Chew F.T. (2020). Systematic review of the epidemiology of acne vulgaris. Sci. Rep..

[12] Kanitakis J. (2002). Anatomy, histology and immunohistochemistry of normal human skin. Eur. J. Dermatol..

[13] Zouboulis C.C. (2000). Human Skin: An Independent Peripheral Endocrine Organ. Horm. Res. Paediatr..

[14] Garcia-Reyero N. (2018). The clandestine organs of the endocrine system. Gen. Comp. Endocrinol..

[15] Zouboulis C.C. (2004). The human skin as a hormone target and an endocrine gland. Hormones.

[16] Zouboulis C.C., Degitz K. (2004). Androgen action on human skin—From basic research to clinical significance. Exp. Dermatol..

[17] Pace S., Werz O. (2020). Impact of Androgens on Inflammation-Related Lipid Mediator Biosynthesis in Innate Immune Cells. Front. Immunol..

[18] Traish A., Bolanos J., Nair S., Saad F., Morgentaler A. (2018). Do Androgens Modulate the Pathophysiological Pathways of Inflammation? Appraising the Contemporary Evidence. J. Clin. Med..

[19] Zhang R., Zhou L., Lv M., Yue N., Fei W., Wang L., Liu Z., Zhang J. (2022). The Relevant of Sex Hormone Levels and Acne Grades in Patients with Acne Vulgaris: A Cross-Sectional Study in Beijing. Clin. Cosmet. Investig. Dermatol..

[20] Gratton R., Del Vecchio C., Zupin L., Crovella S. (2022). Unraveling the Role of Sex Hormones on Keratinocyte Functions in Human Inflammatory Skin Diseases. Int. J. Mol. Sci..

[21] Thiboutot D., Gilliland K., Cong Z., Jabara S., McAllister J.M., Sivarajah A., Clawson G. (2003). Human Skin is a Steroidogenic Tissue: Steroidogenic Enzymes and Cofactors Are Expressed in Epidermis, Normal Sebocytes, and an Immortalized Sebocyte Cell Line (SEB-1). J. Investig. Dermatol..

[22] Zawiślak B., Marchlewicz M., Świder-Al-Amawi M., Wenda-Rózewicka L., Wiszniewska B. (2010). Skóra i jej udział w syntezie hormonów steroidowych. Postep. Biol. Komórki.

[23] Poreba R., Debski R., Kotarski J., Paszkowski T., Pertyn T., Stachowiak G. (2012). Complex hormonal therapy in women with acne—Recommendations of the polish gynecologic society expert panel-2011. Ginekol. Pol..

[24] Akpolat D. (2021). Unexpected Effects of Oral Isotretinoin in Adolescents With Acne Vulgaris. Cureus.

[25] Chilicka K., Rogowska A.M., Szyguła R., Rusztowicz M., Nowicka D. (2022). Efficacy of Oxybrasion in the Treatment of Acne Vulgaris: A Preliminary Report. J. Clin. Med..

[26] Chilicka K., Rusztowicz M., Rogowska A.M., Szyguła R., Asanova B., Nowicka D. (2022). Efficacy of Hydrogen Purification and Cosmetic Acids in the Treatment of Acne Vulgaris: A Preliminary Report. J. Clin. Med..

[27] Burke B.M., Cunliffe W.J. (1984). The assessment of acne vulgaris—The Leeds technique. Br. J. Dermatol..

[28] Slayden S.M., Moran C., Sams W., Boots L.R., Azziz R. (2001). Hyperandrogenemia in patients presenting with acne. Fertil. Steril..

[29] Timpatanapong P., Rojanasakul A. (1997). Hormonal Profiles and Prevalence of Polycystic Ovary Syndrome in Women with Acne. J. Dermatol..

[30] Bunker C., Newton J.A., Kilborn J., Patel A., Conway G., Jacobs H., Greaves M., Dowd P.M. (1989). Most women with acne have polycystic ovaries. Br. J. Dermatol..

[31] Levell M., Cawood M., Burke B., Cunliffe W. (1989). Acne is not associated with abnormal plasma androgens. Br. J. Dermatol..

[32] Lucky A.W., Biro F.M., Simbartl L.A., Morrison J.A., Sorg N.W. (1997). Predictors of severity of acne vulgaris in young adolescent girls: Results of a five-year longitudinal study. J. Pediatr..

[33] Lucky A.W., Cullen S.I., Funicella T., Jarratt M.T., Jones T., Reddick M.E. (1998). Double-blind, vehicle-controlled, multicenter comparison of two 0.025% tretinoin creams in patients with acne vulgaris. J. Am. Acad. Dermatol..

[34] Harper J.C. (2008). Evaluating hyperandrogenism: A challenge in acne management. J. Drugs Dermatol..

[35] Walton S., Cunliffe W., Keczkes K., Early A., McGarrigle H., Katz M., Reese R. (1995). Clinical, ultrasound and hormonal markers of androgenicity in acne vulgaris. Br. J. Dermatol..

[36] Cibula D., Hill M., Vohradnikova O., Kuzel D., Fanta M., Zivny J. (2000). The role of androgens in determining acne severity in adult women. Br. J. Dermatol..

[37] Cannavò S.P., Vaccaro M., Guarneri B., Borgia F., Cannavò S., Guarneri F. (2004). Correlation between Endocrinological Parameters and Acne Severity in Adult Women. Acta Derm. Venereol..

[38] Koulianos G., Thorneycroft I., Schlaff W.D., Rock J.A. (1993). Abnormal sex hormone-binding globulin. Decision-Making in Reproductive Endocrinology and Infertility.

[39] Kurnaz-Gomleksiz O., Akadam-Teker B., Bugra Z., Omer B., Yilmaz-Aydogan H. (2019). Genetic polymorphisms of the SHBG gene can be the effect on SHBG and HDL-cholesterol levels in Coronary Heart Disease: A case–control study. Mol. Biol. Rep..

[40] Xita N., Tsatsoulis A. (2010). Genetic variants of sex hormone-binding globulin and their biological consequences. Mol. Cell. Endocrinol..

[41] Lolis M.S., Bowe W.P., Shalita A.R. (2009). Acne and Systemic Disease. Med. Clin. N. Am..

[42] Imperato-McGinley J., Gautier T., Cai L.Q., Yee B., Epstein J., Pochi P. (1993). The androgen control of sebum production. Studies of subjects with dihydrotestosterone deficiency and complete androgen insensitivity. J. Clin. Endocrinol. Metab..

[43] Tehrani F.R., Behboudi-Gandevani S., Yarandi R.B., Naz M.S.G., Carmina E. (2021). Prevalence of acne vulgaris among women with polycystic ovary syndrome: A systemic review and meta-analysis. Gynecol. Endocrinol..

[44] Carmina E., Lobo R. (2001). Hirsutism, alopecia, and acne. Principles and Practice of Endocrinology and Metabolism.

[45] Reingold S.B. (1987). The Relationship of Mild Hirsutism or Acne in Women to Androgens. Arch. Dermatol..

[46] Carmina E. (2020). Cutaneous manifestations of polycystic ovary syndrome. Curr. Opin. Endocr. Metab. Res..

[47] Sardana K., Bansal P., Sharma L.K., Garga U.C., Vats G. (2020). A study comparing the clinical and hormonal profile of late onset and persistent acne in adult females. Int. J. Dermatol..

[48] Carmina E., Dreno B., Lucky W.A., Agak W.G., Dokras A., Kim J.J., Lobo R.A., Tehrani F.R., Dumesic D. (2022). Female Adult Acne and Androgen Excess: A Report From the Multidisciplinary Androgen Excess and PCOS Committee. J. Endocr. Soc..

[49] Gáspár E., Hardenbicker C., Bodó E., Wenzel B., Ramot Y., Funk W., Kromminga A., Paus R. (2010). Thyrotropin releasing hormone (TRH): A new player in human hair-growth control. FASEB J..

[50] Foitzik K., Krause K., Conrad F., Nakamura M., Funk W., Paus R. (2006). Human Scalp Hair Follicles Are Both a Target and a Source of Prolactin, which Serves as an Autocrine and/or Paracrine Promoter of Apoptosis-Driven Hair Follicle Regression. Am. J. Pathol..

[51] Langan E.A., Hinde E., Paus R.R. (2018). Prolactin as a candidate sebotrop(h)ic hormone?. Exp. Dermatol..

[52] Clayton R.W., Langan E.A., Ansell D., de Vos I., Göbel K., Schneider M.R., Picardo M., Lim X., Van Steensel M.A.M., Paus R. (2020). Neuroendocrinology and neurobiology of sebaceous glands. Biol. Rev..

[53] Cussen L., McDonnell T., Bennett G., Thompson C.J., Sherlock M., O’Reilly M.W. (2022). Approach to androgen excess in women: Clinical and biochemical insights. Clin. Endocrinol..

[54] Sheely D., Pujare D. (2022). Endocrinopathies. Med. Clin. N. Am..

